# High intensity training during spaceflight: results from the NASA Sprint Study

**DOI:** 10.1038/s41526-020-00111-x

**Published:** 2020-08-18

**Authors:** Kirk L. English, Meghan Downs, Elizabeth Goetchius, Roxanne Buxton, Jeffrey W. Ryder, Robert Ploutz-Snyder, Mark Guilliams, Jessica M. Scott, Lori L. Ploutz-Snyder

**Affiliations:** 1 University of Houston-Clear Lake, Houston, TX USA; 2 Health and Human Performance Institute, Houston, TX USA; 3 NASA Johnson Space Center, Houston, TX USA; 4 University of Houston, Houston, TX USA; 5 KBR, Houston, TX USA; 6 University of Michigan, Ann Arbor, MI USA; 7 Memorial Sloan Kettering Cancer Center, New York, NY USA

**Keywords:** Physiology, Outcomes research

## Abstract

Historically, International Space Station (ISS) exercise countermeasures have not fully protected astronauts’ musculoskeletal and cardiorespiratory fitness. Although these losses have been reduced on more recent missions, decreasing the time required to perform in-flight exercise would permit reallocation of that time to other tasks. To evaluate the effectiveness of a new training prescription, ISS crewmembers performed either the high intensity/lower volume integrated Sprint resistance (3 d wk^−1^) and aerobic (interval and continuous workouts, each 3 d wk^−1^ in alternating fashion) exercise program (*n* = 9: 8M/1F, 48 ± 7 y, 178 ± 5 cm, 77.7 ± 12.0 kg) or the standard ISS countermeasure consisting of daily resistance and aerobic exercise (*n* = 17: 14M/3F, 46 ± 6 y, 176 ± 6 cm, 80.6 ± 10.5 kg) during long-duration spaceflight. Bone mineral density (dual energy X-ray absorptiometry (DXA)), muscle strength (isokinetic dynamometry), muscle function (cone agility test), and cardiorespiratory fitness (VO_2peak_) were assessed pre- and postflight. Mixed-effects modeling was used to analyze dependent measures with alpha set at *P* < 0.05. After spaceflight, femoral neck bone mineral density (−1.7%), knee extensor peak torque (−5.8%), cone agility test time (+7.4%), and VO_2peak_ (−6.1%) were decreased in both groups (simple main effects of time, all *P* < 0.05) with a few group × time interaction effects detected for which Sprint experienced either attenuated or no loss compared to control. Although physiologic outcomes were not appreciably different between the two exercise programs, to conserve time and optimally prepare crewmembers for the performance of physically demanding mission tasks, high intensity/lower volume training should be an indispensable component of spaceflight exercise countermeasure prescriptions.

## Introduction

Exercise countermeasures are employed during spaceflight to combat the deleterious physiologic effects of long-duration microgravity exposure. Early exercise hardware on the International Space Station (ISS) was limited to low running velocities (treadmill with vibration isolation system, TVIS: ~11.3 km h^−1^ peak permitted velocity) and low loads (interim resistive exercise device, iRED: 136 kg peak load), making a low intensity/high volume exercise program the default prescription^1^. Despite the near-daily performance of this program, multisystem deconditioning was evident including decrements in muscle mass, strength, and function^2–5^, cardiorespiratory fitness^6^, and bone mineral density^7,8^.

The effectiveness of high intensity/low volume training (HIT) has been extensively documented in populations ranging from elite athletes^9^ to clinical patients^10,11^. In addition to the time savings of shorter exercise sessions, there is evidence to suggest that HIT may elicit superior physiologic adaptations compared to traditional lower intensity/higher volume training. For instance, over a 6-week period (5 d wk^−1^), Tabata et al.^12^ compared 60 min bouts of continuous exercise (70% VO_2peak_) to 7–8 intervals (20 s at 170% VO_2peak_/10 s rest). Despite cumulative exercise time of only ~2 h compared to 30 h for the continuous group, the HIT group increased both aerobic and anaerobic capacity whereas the continuous, high volume group only improved aerobic capacity. Somewhat longer-duration intervals of 2–4 min have been shown to maintain or improve aerobic capacity during bed rest unloading^13,14^ and athletic training^9^. High intensity training also has been employed in resistance exercise and elicits superior increases in muscle mass^15^ and strength compared to lower intensities with equivalent volume load^15^.

To facilitate higher intensity aerobic and resistance exercise, the original suite of ISS exercise hardware was replaced in 2009 with a second-generation treadmill (T2: 19.3 km h^−1^ peak velocity) and the advanced resistive exercise device (ARED: 272 kg peak load)^16^. However, power issues initially limited T2’s peak velocity to 14.5 km h^−1^ ^17^ and although crewmembers could lift heavier loads on ARED (up to 6-repetition maximum loads have been used based on crewmember capability and preference)^1^, they continued to perform both aerobic and resistance exercise 6 days per week. This approach consumed 9–10 h per week^1,5^, a significant time commitment that reduced crewmembers’ availability to perform other important mission tasks.

Thus, in light of the potential for similar, if not superior, physiologic protection coupled with meaningful time savings, the purpose of this investigation was to compare physiologic outcomes after ~6 months of long-duration spaceflight in crewmembers who performed exercise countermeasures consisting of either (1) lower intensity/higher volume exercise (6 d wk^−1^ resistance exercise and 6 d wk^−1^ aerobic exercise) or (2) high intensity/lower volume exercise (3 d wk^−1^ resistance exercise and 6 d wk^−1^ aerobic exercise). The study’s original hypothesis was that the experimental exercise protocol (Sprint) would better mitigate spaceflight-induced musculoskeletal and cardiorespiratory deconditioning compared to standard of care ISS exercise (Control). Over time, the culture surrounding high intensity exercise during spaceflight changed. Early successes with Sprint, lack of adverse effects, improved pre-flight cardiovascular risk screening, and other factors responsibly led to an evolution of the standard of care. This study is an exemplar of how research should transition to operations; this transition was likely accelerated by having operations colleagues on the research team. This rapid operational success story does pose a unique challenge to the scientific interpretation of the data; we readily acknowledge the complexity of this situation and the limitation of the evolving control group.

## Results

### Exercise training

SPRINT subjects effectively performed the aerobic exercise protocol as mean, peak heart rates for all intervals performed were >90% HR_max_ (Supplementary Table 1). Compared to CON, in-flight aerobic exercise volume for SPRINT was 29% and 7% lower on the cycle ergometer with vibration isolation system (CEVIS) and T2, respectively; total combined in-flight aerobic exercise volume was 17% lower for SPRINT. Aerobic exercise intensity was similar between CON and SPRINT for select interval and continuous workouts that each group performed. In-flight resistance exercise average loads for squat, heel raise, and deadlift were 6–15% higher in SPRINT while repetitions per week were 41–46% less for SPRINT. Total weekly resistance exercise volume load (sets × repetitions × load) was 34–44% lower for SPRINT (Supplementary Table 2).

### Bone mineral density

Bone mineral density of the lumbar spine, pelvis, total hip, trochanter, femoral neck, and calcaneus were reduced after spaceflight (simple main effect for time, *P* < 0.05; Table 1). No group × time interactions were detected.

**Table 1:** Bone mineral density and leg lean and fat tissue mass before and after long-duration spaceflight with standard of care exercise or Sprint exercise prescription.

	CON		SPRINT	
	Preflight	Postflight	Preflight	Postflight
Lumbar spine (g cm^−2^)	1.088 ± 0.024	1.068 ± 0.024*	1.067 ± 0.032	1.053 ± 0.032*
Pelvis (g cm^−2^)	1.289 ± 0.034	1.247 ± 0.035*	1.249 ± 0.047	1.208 ± 0.047*
Total hip (g cm^−2^)	1.054 ± 0.026	1.023 ± 0.026*	1.045 ± 0.036	1.027 ± 0.036*
Trochanter (g cm^−2^)	0.797 ± 0.023	0.771 ± 0.023*	0.786 ± 0.032	0.774 ± 0.032*
Femoral neck (g cm^−2^)	0.860 ± 0.025	0.846 ± 0.025*	0.862 ± 0.034	0.845 ± 0.034*
Both legs (g cm^−2^)	1.328 ± 0.026	1.323 ± 0.026	1.260 ± 0.036	1.247 ± 0.036
Calcaneus (g cm^−2^)	0.729 ± 0.025	0.722 ± 0.025*	0.677 ± 0.034	0.666 ± 0.034*
Leg lean mass (kg)	18.77 ± 0.68	18.64 ± 0.68	18.37 ± 0.94	18.28 ± 0.04
Leg fat mass (kg)	6.10 ± 0.43	5.75 ± 0.43*	6.02 ± 0.62	5.69 ± 0.62*

Data are mean ± SE and were collected via DXA approximately 90 days preflight and 7–14 days postflight. No significant group × time interaction effects were detected (*P* > 0.05). *CON* control group that performed the ISS standard of care exercise prescription, *SPRINT* experimental group that performed a high intensity/lower volume exercise prescription.

*Simple main effect for time relative to preflight (*P* < 0.05).

### Muscle mass

Leg lean mass was unchanged after spaceflight (simple main effect for time, *P* = 0.40; Table 1) and there was no group × time interaction (*P* = 0.88; Table 1). Leg fat mass was decreased after spaceflight (simple main effect for time, *P* = 0.001; Table 1) but there was no group × time interaction.

### Muscle strength

At the first post-flight test (*R* + 5), all tested isokinetic peak torque and total work variables were decreased (simple main effect for time, *P* < 0.05; Table 2). On *R* + 14, this reduction persisted for all variables (simple main effect for time, *P* < 0.05; Table 2) except eccentric ankle plantar flexor peak torque (*P* = 0.06; Table 2). At *R* + 30 testing, only knee extensor and flexor peak torque and knee flexor total work still differed significantly from preflight baseline (simple main effect for time, *P* < 0.05; Table 2). Group × time interaction effects were present for knee flexor peak torque (*R* + 5, *R* + 14, and *R* + 30, each *P* < 0.05; Table 2) and trunk extensor peak torque (*R* + 14, *P* = 0.01; Table 2); for these interactions, reductions in strength were absent or attenuated in SPRINT.

**Table 2:** Isokinetic muscle strength and endurance before and after long-duration spaceflight with standard of care exercise or Sprint exercise prescription.

	CON				SPRINT			
	Preflight	*R* + 5	*R* + 14	*R* + 30	Preflight	*R* + 5	*R* + 14	*R* + 30
Knee ext, 60° s^−1^	208 ± 11	194 ± 11*	191 ± 11*	195 ± 11*	191 ± 15	177 ± 15*	186 ± 11*	188 ± 15*
Knee flex, 60° s^−1^	113 ± 6	102 ± 6***	103 ± 6*,**	107 ± 6*,**	100 ± 7	100 ± 7*	102 ± 7*	103 ± 7*
Knee ext, 180° s^−1^	2657 ± 121	2466 ± 121***	2509 ± 121*	2599 ± 121	2500 ± 161	2207 ± 161*	2381 ± 161*	2406 ± 161
Knee flex, 180° s^−1^	1573 ± 77	1403 ± 77^*^	1439 ± 77*	1489 ± 77*	1518 ± 103*	1394 ± 103*	1461 ± 103*	1515 ± 103*
Ankle conc PF, 30° s^−1^	134 ± 6	120 ± 6*	123 ± 6*	132 ± 6	131 ± 8	116 ± 8*	120 ± 8*	133 ± 8
Ankle ecc PF, 30° s^−1^	191 ± 11	172 ± 11*	180 ± 11	189 ± 11	193 ± 15	161 ± 15*	174 ± 15	198 ± 15
Trunk ext, 60° s^−1^	446 ± 26	–	408 ± 27*,**	423 ± 27	400 ± 35	–	429 ± 36*	393 ± 35
Trunk flex, 60° s^−1^	225 ± 10	–	196 ± 12*	210 ± 11	196 ± 14	–	172 ± 14*	181 ± 14

Data are mean ± SE and represent peak torque for the single highest repetition (Nm) or total work for an entire set of repetitions (J; knee extension and flexion at 180° s^−1^) during isokinetic dynamometry conducted approximately 50 days preflight (L-50) and 5, 14, and 30 days postflight (*R* + 5, *R* + 14, and *R* + 30). *Per protocol* trunk strength is not assessed at *R* + 5 testing, *CON* control group that performed the ISS standard of care exercise prescription, *SPRINT* experimental group that performed a high intensity/lower volume exercise prescription, *ext* extension, *flex* flexion, *conc PF* concentric plantarflexion, *ecc PF* eccentric plantarflexion.

*Simple main effect for time relative to preflight (*P* < 0.05).

**Simple group × time interaction effect relative to preflight (*P* < 0.05).

### Muscle function

Leg press 1RM was unchanged after spaceflight (*R* + 7, simple main effect for time, *P* > 0.05; Table 3); at *R* + 30, strength was significantly greater than preflight baseline (simple main effect for time, *P* = 0.04; Table 3). Bench press 1RM was increased from preflight at both *R* + 7 and *R* + 30 (simple main effects for time, *P* < 0.05; Table 3). No interaction effects were detected for either 1RM test. Flexibility was decreased at *R* + 7 (simple main effect for time, *P* < 0.01; Table 3); a group × time interaction effect was also present with CON losing flexibility to a greater extent than SPRINT (*P* = 0.01; Table 3). Time to complete the cone test was increased at *R* + 7 (simple main effect for time, *P* < 0.01; Table 3); a trend for a group × time interaction effect was also present with CON tending to increase time to completion more than SPRINT (*P* = 0.07; Table 3).

**Table 3:** Functional muscle performance and flexibility before and after long-duration spaceflight with standard of care exercise or Sprint exercise prescription.

	CON			SPRINT		
	Preflight	*R* + 7	*R* + 30	Preflight	*R* + 7	*R* + 30
Leg press 1RM (kg)	304 ± 18	299 ± 18	317 ± 18*	284 ± 22	270 ± 22	289 ± 22*
Bench press 1RM (kg)	88 ± 6	91 ± 6*	94 ± 6*	79 ± 7	82 ± 7*	85 ± 7*
Sit and Reach (cm)	17.4 ± 0.9	16.3 ± 0.9*,**	17.8 ± 0.9	18.2 ± 1.1	18.1 ± 1.1*	18.9 ± 1.1
Cone test (s)	12.5 ± 0.2	13.5 ± 0.3*	12.3 ± 0.2	12.7 ± 0.3	13.1 ± 0.3*	12.4 ± 0.3

Data are mean ± SE and were collected approximately 60–90 days preflight (L-60) and 7 and 30 days postflight (*R* + 7 and *R* + 30). *CON* control group that performed the ISS standard of care exercise prescription, *SPRINT* experimental group that performed a high intensity/lower volume exercise prescription.

*Simple main effect for time relative to preflight (*P* < 0.05).

**Simple group × time interaction effect relative to preflight (*P* < 0.05).

### Cardiorespiratory fitness

Absolute and relative VO_2peak_ were decreased on *R* + 1/*R* + 3 (simple main effect for time, both *P* < 0.05; Table 4). Similarly, ventilatory threshold and peak workload were reduced immediately postflight (*R* + 1/*R* + 3, simple main effect for time, both *P* < 0.01; Table 4). HR_peak_ was unchanged after spaceflight. No group × time interaction effects were detected for any cardiorespiratory outcomes.

**Table 4:** Cardiorespiratory parameters before and after long-duration spaceflight with standard of care exercise or Sprint exercise prescription.

	CON			SPRINT		
	Preflight	*R* + 3	*R* + 30	Preflight	*R* + 1	*R* + 30
VO_2peak_ (L min^−1^)	3.36 ± 0.19	3.12 ± 0.19*	3.32 ± 0.19	3.22 ± 0.23	2.91 ± 0.23*	3.23 ± 0.23
VO_2_peak (mL kg^−1^ min^−1^)	41.8 ± 1.6	39.7 ± 1.6*	41.8 ± 1.6	41.6 ± 1.9	38.2 ± 1.9*	42.0 ± 1.9
Ventilatory threshold (L min^−1^)	2.22 ± 0.15	1.97 ± 0.15*	2.25 ± 0.15	2.08 ± 0.16	1.79 ± 0.18*	2.00 ± 0.18
Peak workload (W)	302 ± 14	281 ± 14*	304 ± 14	269 ± 17	256 ± 17*	288 ± 17
Peak heart rate (beats min^−1^)	173 ± 3	174 ± 3	174 ± 3	178 ± 4	176 ± 4	176 ± 4

Data are mean ± SE and were collected approximately 50 days preflight (L-50) and 1 (Sprint) or 3 (CON) days and 30 days postflight (*R* + 1/*R* + 3 and *R* + 30). No group × time interaction effects were detected (*P* > 0.05). *CON* control group that performed the ISS standard of care exercise prescription, *SPRINT* experimental group that performed a high intensity/lower volume exercise prescription.

*Simple main effect for time relative to preflight (*P* < 0.05).

## Discussion

**Fig. 1:**
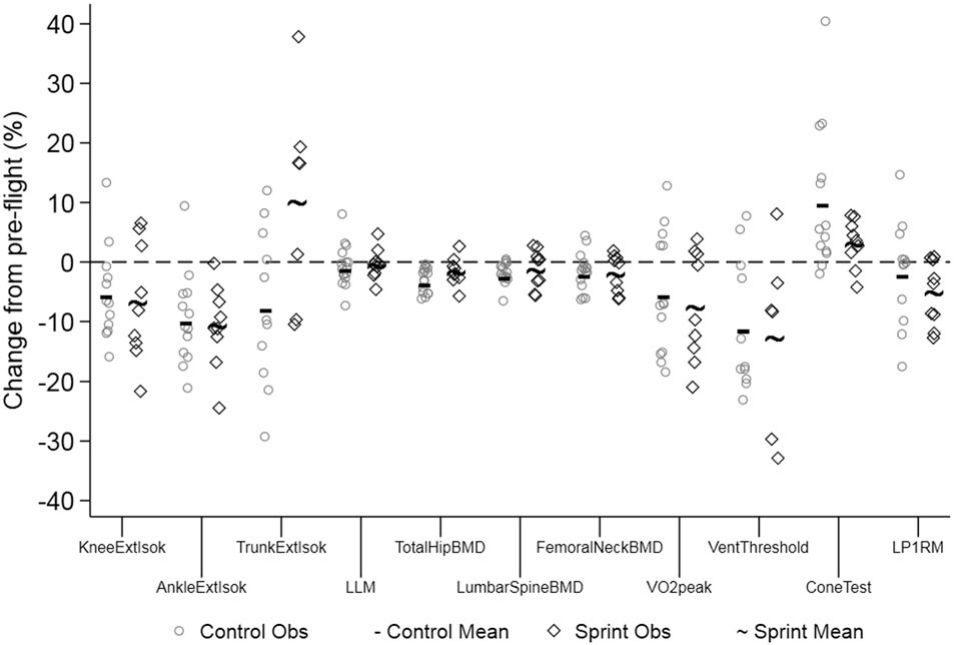
Spaceflight-induced multisystem physiologic and functional alterations. Individual (open symbols) and mean (solid marks) percent losses from last preflight to first postflight test for control (open circles; hyphens) and Sprint subjects (open squares; tildes) for selected bone mineral density, muscle mass, muscle strength, muscle function, and cardiorespiratory fitness parameters. KneeExtIsok knee extension isokinetic peak torque at 60°s^−1^, AnkleExtIsok ankle extension isokinetic peak torque at 30°s^−1^, TrunkExtIsok trunk extension isokinetic peak torque at 60°s^−1^, LLM lean leg mass, BMD bone mineral density, VentThreshold ventilatory threshold, LP1RM leg press 1-repetition maximum. *Observations were excluded if not in the model, or missing pre or post.

We evaluated the protective effects of a high intensity/lower volume integrated aerobic and resistance exercise countermeasure to the multisystem deconditioning of long-duration spaceflight aboard the ISS; we compared these results to those of the somewhat lower intensity/higher volume regimen that is the standard exercise protocol for ISS crewmembers^1^. Overall, we found significant decrements in bone mineral density, muscle strength and endurance, and cardiorespiratory performance after long-duration spaceflight; these changes were mostly independent of the exercise countermeasure that was performed (Fig. 1).

In these results are a few noteworthy findings that have important application for astronauts’ mission critical task performance (e.g., extravehicular activity). First, the relative change in ventilatory threshold was approximately double that of VO_2peak_; this is similar to previous findings in bed rest^18^. Ventilatory threshold and other defined submaximal work levels (e.g., lactate threshold) have been shown to be more predictive of athletic performance than VO_2peak_^19,20^; this raises the interesting possibility that ventilatory threshold may be a better parameter for monitoring aerobic fitness in astronauts because it is more sensitive to change, is more aligned with the submaximal nature of occupational work, and may be a better indicator of fitness for duty (i.e., performance). Second, trunk extension strength (Table 2) was notably improved in Sprint subjects which may be related to the higher resistance exercise training intensity. Preservation of lumbar muscle strength may be helpful for prevention of spaceflight-induced back pain or disc herniation^21,22^.

High intensity training, while novel to spaceflight when this study was first implemented, is not a new phenomenon. It has been studied extensively in clinical^23,24^, community-dwelling^25^, and athletic populations^9^; these populations range in age from young children^26,27^ to older adults^28,29^. We and others have evaluated HIT in bed rest^14,30^, a ground-based analog of spaceflight, where it is largely effective to prevent multisystem physiologic deconditioning^31^. The appeal of HIT hinges on two potential benefits: (1) superior physiologic adaptations compared to traditional, longer-duration steady state/lower intensity training and (2) shorter/less frequent training sessions that offer different and distinct advantages for each of the aforementioned populations.

Analysis of exercise training logs revealed that aerobic exercise volume was slightly greater in CON while aerobic intensity—at least for a subset of common interval workouts—was similar between the Sprint and control groups; both groups performed aerobic exercise 6 days per week using a combination of interval and continuous workouts. Before and during the earliest part of this study (2011), aerobic interval exercise was employed rarely in the ISS standard care exercise prescription (CON) due to hardware limitations, but by the end of the project (2017) it had become an integral and regular component of the standard aerobic exercise regimen. Although this complicates the research design, it is increasingly important, especially in complex interdisciplinary research that spans many years, to develop methods of accepting and interpreting data when “standard of care” may change over the course of an investigation. In aggregate, our findings show similarity between the current individualized standard care exercise countermeasure and the high intensity/lower volume Sprint protocol with regards to spaceflight-induced changes in bone mineral density, muscle mass, muscle strength and function, and cardiorespiratory fitness. Based on these metrics alone, one would reasonably argue that the two programs are interchangeable. This can be viewed positively as providing flexibility for exercise programming: while some crewmembers may enjoy higher intensity/lower volume exercise such as the Sprint prescription, others may prefer the somewhat lower intensity/higher volume program.

Despite similar physiologic outcomes, the two programs differ in their requirements for one particularly valuable and limited resource: time. Of the current 2.5 h scheduled for the performance of 6 d wk^−1^ exercise countermeasures, ~60 min (45–75 min) is allocated to resistance exercise and ~30–45 min devoted to aerobic exercise for a total daily exercise duration of 1.5–2.0 h^1,5^. The low end of this range (1.5 h × 6 d wk^−1^) equates to a weekly exercise total of 9 h. In contrast, Sprint consumes at least 33% less time than the standard exercise program: three 60-min resistance exercise sessions, three 30-min continuous sessions, plus 3 aerobic interval sessions totaling <90 min equals ~6 h^31^. For future exploration missions with compressed timelines, small cabin spaces, and likely a single exercise device on which to perform both aerobic and resistance exercise for an entire crew, brief high intensity exercise prescriptions will be an extremely attractive programming option due to their efficacy and efficiency. Our bed rest studies have demonstrated that the Sprint exercise prescription is similarly effective over a range of exercise modalities and equipment including traditional ISS-like exercise equipment with or without testosterone supplementation and using only a single compact single exercise device that combines rowing and resistance exercise^31^.

Resistance exercise differed most markedly between the groups on its frequency. Sprint subjects performed about half of the number of repetitions on ARED, which is important both from a time and equipment usage/maintenance perspective. If equipment availability were to change in the future, or there was concern about wear and tear on exercise equipment, 3 days per week of resistance exercise, if intensity is maintained at a high level, should offer sufficient protection as evidenced by the mostly similar musculoskeletal outcomes we observed in the two groups despite markedly different total volume loads (sets × repetitions × load).

It is extremely important to note that physiologic outcomes across systems in both study groups (pooled) were substantially better than those previously reported in ISS crewmembers. The most modest differences were observed for muscle strength variables. For example, declines in isokinetic strength about the knee (−6%), ankle (−10%), and trunk (−5%) for the present study were notably better than previous crewmembers who exercised with iRED (knee: −14%, ankle: −14%, trunk: −7%) and moderately better compared to the losses of early ARED users (knee: −7%, ankle: −13%, trunk: −5%)^2^. Although leg press 1RM losses (−3%) were comparable to previously reported ISS values (−3%), the more functional cone test time (+7%) was better preserved than in previous crewmembers (+11%)^32^.

The greatest improvements were observed in bone health and cardiovascular fitness. Bone mineral density losses from the Russian Mir station, which lacked a resistance exercise capability, were 1.06%/month (lumbar spine) and 1.15%/month (femoral neck)^33^. When extrapolated to multi-month missions, such losses were alarming. Incremental improvements were made with the addition of iRED resistance exercise on the ISS (−3.7% lumbar spine; −6.1% femoral neck over ~6-month missions)^34^. Further improvement was observed after the installation of ARED (−2.6% lumbar spine; −4.1% femoral neck)^34^. The data reported in this paper of −1.7% (lumbar spine) and −1.7% (femoral neck) over multi-month missions represent another improvement. Such losses are within the precision error of the DXA measurement and can be considered negligible. It remains possible that changes in bone architecture (not evaluated in this report) could still exist, but bone density is now reasonably maintained.

A similar success story is observed for cardiorespiratory fitness. For the cardiorespiratory system, we measured a 6% decrease in VO_2peak_ from preflight to postflight while our previous study of ISS crewmembers found a ~15% decrement in aerobic capacity postflight^35^. Together, these data suggest that current ISS exercise countermeasures (both standard of care and Sprint) provide considerably better protection of musculoskeletal and cardiorespiratory outcomes during long-duration spaceflight than did previous hardware and training protocols. These excellent outcomes allow us to consider, for the first time, whether the exercise countermeasures are sufficient or whether additional optimization is necessary. This raises interesting questions: Do we need to completely mitigate in-flight loss? If not, how much loss is acceptable? Does the amount of loss that would be accepted depend, at least in part, on the initial starting point for that individual or the mission tasks or landing scenarios that individual will be asked to perform? It is now time to shift the paradigm to consider these individual details and view astronauts as a tactical population akin to the military, police, and firefighters^36^. These operational professions have evolved from endeavoring to simply maintain an arbitrary fitness threshold to testing and preparing personnel to ensure that they are physiologically capable of meeting the demands of their job. In the last 5 years, both the Canadian Armed Forces and the US Army have completely overhauled their fitness for duty standards; both retired test batteries that largely featured tests of muscle and aerobic endurance and in their place adopted standards that inclusively evaluate aerobic fitness, anaerobic fitness, and muscle strength/power^37,38^. These changes were motivated by the inability of the previous fitness tests to predict performance in the field. In this new perspective that tightly links and subordinates testing and training to job performance, it is easy to envision a primary role for HIT in the preparation and training of soldiers and astronauts alike. Indeed, the developers of these new military fitness standards highlight the direct applicability of high intensity/low volume interval training (in contrast to legacy training that centered on low intensity/high volume exercise) to enhanced health and performance in military personnel^39^.

To fully extend this paradigm to astronauts and long-duration spaceflight, we must consider the key functions of exercise countermeasures that are essentially threefold: (1) to prevent long-term negative health consequences, (2) to ensure safe return to Earth, and (3) to facilitate optimal performance and the achievement of critical mission objectives. The two latter purposes are particularly salient to the “tactical population” view and the role of high intensity interval and strength training in astronaut fitness for duty. Return to Earth from the ISS is currently accomplished via the Russian Soyuz capsule in a well-supported terrestrial landing operation that requires relatively little physiologic effort of returning crews (e.g., they are physically extracted from the capsule). Upcoming flights aboard new commercial and NASA spacecraft will splashdown in the ocean with vehicle recovery and crew extraction nominally performed by the U.S. Navy and Air Force. However, in a contingency situation (e.g., an off target splashdown and/or a cabin emergency necessitating immediate evacuation), crewmembers will be required to egress the capsule unaided into a small, tethered life raft. Not surprisingly, preliminary testing with suited human subjects indicates that the physiologic demand of this relatively brief, off-nominal operation is moderate to high (unpublished data). Although unlikely, this operation represents a potential scenario for which crewmembers must be physiologically prepared upon return to Earth’s 1*g* environment. Much more certain—if not further into the future—are terrestrial exploration missions that will include unaided egress and a host of demanding surface operations (e.g., hill climbs and descents, habitat construction, materials transfer, and geologic equipment operation); potential off-nominal tasks include incapacitated crewmate rescue^40,41^. The functional relevance and metabolic specificity of high intensity resistance and aerobic interval exercise to the performance of critical mission tasks suggest that HIT should be a key component of astronaut exercise training and countermeasures.

This investigation has several limitations. First, astronauts self-selected into the group of their choosing. This was necessary because of the voluntary nature of spaceflight research studies as well as crewmembers’ reluctance to relinquish control of the fundamental nature of their exercise programs especially early in the study. Second, as with many spaceflight studies, the investigation has a relatively low number of participants, increasing the likelihood of a Type II error. Third, by its very nature, the standard care exercise countermeasure program employed by CON subjects included a degree of individualization and temporal change; this is in contrast to the Sprint protocol which was an inflexible experimental protocol. As previously noted, due to the study’s operational environment, the standard care exercise prescription evolved over the ~6 y study duration to include higher aerobic exercise intensities. Finally, not all post-flight testing was completed at the same time; for instance, although we were able to perform VO_2peak_ tests for experimental subjects (SPRINT) on *R* + 1, most CON subjects were tested on *R* + 3, the typical timeframe for returning ISS crewmembers.

In summary, we evaluated the physiologic effects of a high intensity/lower volume experimental exercise countermeasure compared to the standard higher volume program performed by long-duration ISS crewmembers. Both exercise programs provided substantially better physiologic protection than historic exercise programs, and for current ISS missions, either program is an excellent option. The Sprint training program had outcomes that were as good as or, for a few variables, slightly better than control and offers several distinct advantages: (1) Sprint can be performed in a substantially shorter amount of time with less exercise equipment usage and (2) it may provide a more occupationally specific training experience that will better prepare crewmembers to optimally perform critical mission tasks while remaining safe and healthy.

## Methods

### Overview of research design

All National Aeronautics and Space Administration (NASA), Canadian Space Agency (CSA), European Space Agency (ESA), and Japan Aerospace Exploration Agency (JAXA) astronauts assigned to ISS flight were eligible to participate in this investigation. Subjects self-selected into one of two groups: (1) subjects that performed the experimental Sprint exercise program on the ISS (SPRINT) or (2) subjects that performed the standard individualized exercise program on the ISS (CON); all subjects completed the standard physiologic tests required of ISS crewmembers.

### Subjects and facilities

Testing for this study was performed during ISS Increments 26S–50S (April 2011–September 2017). Twenty-six astronauts assigned to long-duration ISS missions participated (Table 5). Subject enrollment in the two groups was proportionally equivalent across the study such that neither group had a high concentration of subjects during a particular time period. The study was approved by the Institutional Review Board at NASA Johnson Space Center (JSC, Houston, TX), the Japan Aerospace Exploration Agency (JAXA) Institutional Review Board, the European Space Agency (ESA) Medical Board, and the Human Research Multilateral Review Board; all subjects provided written informed consent before participating in the study. The research was conducted in accord with the Declaration of Helsinki. All preflight and postflight testing was performed at NASA JSC. Room temperature was controlled between 20–23 °C. Exercise training during flight was conducted in the U.S. Laboratory Module of the ISS with cabin conditions similar to those on the ground with temperatures ranging from 20 to 22 °C and relative humidity of 30–40%. All crewmembers completed standard preflight medical screening and received clearance from their flight surgeon before participating in the study.

**Table 5:** Subject characteristics.

	CON (*n* = 17; 14M/3F)	SPRINT (*n* = 9; 8M/1F)	All (*N* = 26)
Age (y)	46 ± 6	48 ± 7	47 ± 6
Height (cm)	176 ± 6	178 ± 5	176 ± 6
Weight (kg)	80.6 ± 10.5	77.7 ± 12.0	79.6 ± 11.4
Mission duration (d)^*^	165 ± 42	152 ± 24	160 ± 36

Data are mean ± SD. *CON* control group that performed the ISS standard of care exercise prescription, *SPRINT* experimental group that performed a high intensity/lower volume exercise prescription.

*One subject completed a 340-d mission; that value is not included in the mission duration means; however, the subject’s data are included in all analyses of dependent variables.

### Exercise training

#### Exercise hardware

In-flight aerobic exercise was performed using the second-generation treadmill (T2) and CEVIS; resistance exercise was performed with ARED^17^. T2 was modified from a commercial Woodway Path treadmill (Woodway, Waukesha, WI) to support walking and running exercise between 2.4 and 19.3 km h^−1^. The user is loaded through a shoulder and waist harness that is terminally attached via bungee cords and c-clips to the treadmill deck surface. CEVIS operates similarly to a standard cycle ergometer providing workloads between 25 and 350 W at pedal speeds from 30 to 120 revolutions per minute. Crewmembers wore cycling shoes that snapped into the pedals and strapped themselves with a belt to the CEVIS frame or used the frame handles to remain appropriately positioned on the cycle. ARED simulates free weights with a constant load of 11–272 kg provided by vacuum cylinders and an inertial load effected by flywheels placed in the load path; both barbell and cable exercises can be performed^16^.

#### Exercise prescription

All subjects were matched with a NASA Astronaut Strength, Conditioning, and Rehabilitation (ASCR) specialist approximately two years in advance of their mission. Each subject’s ASCR developed an individualized exercise program based on their group assignment. Subjects also performed preflight exercise training to familiarize themselves with the inflight exercise devices and protcols and for preflight conditioning; exercise programs were not standardized during the preflight phase.

The Sprint exercise prescription is an evidence-based, integrated training program that consists of high intensity, lower volume exercise 6 d wk^−1^ (3 days of resistance and 6 days of aerobic)^31,42,43^. Subjects in this group completed high intensity interval aerobic exercise (3 d wk^−1^) and continuous aerobic exercise (3 d wk^−1^) on alternating days (Supplementary Table 3). Specifically, each of the three interval workouts was completed once per week (8 × 30-s intervals; 6 × 2-min intervals; and 4 × 4-min intervals); continuous aerobic exercise consisted of 30-min bouts at 75% VO_2peak_ (Supplementary Table 3). Aerobic intensities were initially established based on heart rate (HR) at a percentage of VO_2peak_ and HR response to exercise during preflight. Exercise prescriptions were adjusted during flight based on in-flight training, crewmember communication with their ASCR, and in-flight VO_2peak_ cycle tests (not reported here). HR was monitored continuously during training sessions.

Resistance training for Sprint followed an undulating periodized model and was performed on the same day as the continuous aerobic exercise. Most days and when possible, continuous aerobic exercise was performed second, 4–6 h after the resistance exercise session to optimize adaptations. For the typical 6-month mission, resistance training was comprised of a single 24-week mesocycle. After an initial 2-week acclimatization period, load and repetitions were varied on a daily basis (high volume = 4 sets of 12 repetitions, moderate volume = 4 sets of 8 repetitions, low volume = 4 sets of 6 repetitions). Loads were prescribed such that the fourth set of each exercise was performed at near maximal to maximal intensity for the prescribed number of repetitions. Further, subjects were instructed to perform the fourth set of each exercise at each workout to muscle failure with loads for subsequent sessions adjusted accordingly. Thus, all sessions were defined as high intensity with the volume and load varying throughout the week. Subjects rotated among three routines throughout a mission (Supplementary Table 4). Sprint subjects performed upper body resistance exercise similar to that of control subjects (described below) 3 d wk^−1^. Time to perform the Sprint program was approximately 6 h per week (1.5 h continuous, <1.5 h intervals, and 3 h resistance exercise).

Control subjects participated in the standard care exercise countermeasure protocol. The program typically consisted of 1.5–2.0 h per day total of aerobic and resistance exercise, each performed 6 days per week. Although 2.5 h were scheduled for daily exercise on the ISS^1^, typically, exercise time was divided into 30–45 min of aerobic training and 60–75 min of resistance training with hardware configuration and postexercise hygiene comprising the remainder of total allotted time. Aerobic training consisted of interval or continuous steady-state exercise on either CEVIS or T2. The CEVIS protocols were developed using the preflight VO_2peak_ test with prescribed work rates (W) between 70 and 100% VO_2peak_. The ASCRs adjusted the protocols during the mission based on individual performance during training sessions and crew feedback. The T2 protocols were based on pre-flight training and prescribed at 70–100% HR_max_. For most crewmembers, external (harness/bungee) loading began at 60% bodyweight (static load measured when standing stationary on the treadmill belt) and increased to ~75–80% bodyweight as tolerated throughout the mission^1^. Resistance training followed a 9-day periodized program with linear progression of loads and undulating volume across two 12-week mesocycles (Supplementary Table 5). After a 2-week acclimatization period, loads were set at 70% of the repetition-maximum (RM) prescribed for that session (e.g., for a 4 × 6-repetition session, loads in Week 3 were 70% of 6RM) with loading intensity increasing 5% each week. Strength increases over the first mesocycle allowed most crewmembers to reach intensities of 110–120% of their early mission RMs by week 12. For the second mesocycle, loads were reduced to 70% of the crewmember’s new RM (determined from recent training session loads) and the progression of the first mesocycle was repeated. Thus, resistance exercise loading intensity and progression were lower and more conservative, respectively, compared to the Sprint protocol; this was compensated for with twice the workout frequency (6 d wk^−1^ vs. 3 d wk^−1^). A variation of squat, deadlift, and heel raises were each prescribed daily for CON subjects followed by rotating exercises focusing on upper body and stability musculature. Heel raises were prescribed as 4 sets × 20 repetitions. Time to perform the CON program was approximately 9–10 h per week (3–4 h continuous and interval aerobic exercise and 6–7 h resistance exercise).

### Outcome measures

#### Exercise training

Aerobic and resistance exercise training variables were recorded and are presented descriptively. Aerobic exercise outcomes were CEVIS and T2 normalized volume (total min/mission duration in weeks) and average peak HR (b min^−1^ and % maximum) for 30-s, 2-min, and 4-min intervals and 30-min continuous sessions. For resistance exercise, average load (kg) and average repetitions per week were calculated for squat, heel raise, and deadlift and their variations (“squat”: back squat, single leg squat, sumo squat; “heel raise”: heel raise and single leg heel raise; “deadlift”: deadlift, Romanian deadlift, and sumo deadlift). Total volume load (sets × repetitions × load) for each subject’s mission was also calculated and normalized to mission duration (total volume/mission duration in weeks).

#### Bone densitometry and muscle mass

DXA scans were obtained using a single densitometer (Hologic Discovery; Hologic Inc., Waltham, MA, USA). Two bone densitometry technologists, certified by the International Society for Clinical Densitometry (ISCD), performed and analyzed the scans. For a given crewmember, a single technologist performed both the preflight and postflight scans. Scans were performed at approximately 90 days preflight (L-90) and again 1–2 weeks after landing (*R* + 7). At each test session, the following fan-beam DXA scans were performed: left and right hip, lumbar spine, whole body, and left heel. Scans were performed and analyzed according to standard procedures recommended by the manufacturer, with the exception of hip and heel scans. The global region of interest box for the hip was positioned manually, with the lateral margin placed adjacent to the lateral cortex of the greater trochanter and the distal border placed a set number of lines from the lesser trochanter’s distal margin^44,45^. Heel scans were obtained using the forearm scan mode, with the subject seated on the scanner and the foot restrained in a lateral position within a custom jig. In addition to areal bone mineral density (BMD, g cm^2^) obtained from the scans listed above, whole body and regional lean mass (fat-free, bone-free mass) and fat mass were determined from the whole body scans using standard Hologic analysis software. The BMD precision values (% coefficient of variation, % CV) for the scanning laboratory were as follows: left and right total hip, 0.7% and 0.9%; left and right trochanter, 1.1% and 1.1%; left and right femur neck, 1.2% and 1.5%; lumbar spine, 0.5%; heel, 1.0%; and whole body, 1.0%. Precision of soft tissue values from the whole body scans were: whole body lean mass, 0.9% and whole body fat mass, 2.1%. Calibration of the Hologic densitometer was verified by regular scanning of a calibration phantom (at least weekly as well as on the day of subject testing) with scans analyzed using the manufacturer’s automated software.

#### Muscle strength

Isokinetic strength data were acquired for the knee, ankle, and trunk via a Biodex System 4 dynamometer (Biodex Medical Systems, Shirley, NY) using NASA’s Medical Volume B 5.3 Isokinetic Testing protocol^2^. Subjects were tested twice prelaunch (L-9 months and L-50 d) with the latter test used as the preflight baseline and three times after return (*R* + 5 d, *R* + 14 d, and *R* + 30 d). Trunk testing was not performed at the *R* + 5 postflight test as specified in the Medical Volume B 5.3 Isokinetic Testing protocol due to concerns about post-flight low back pain. The right leg was used for all testing unless previous injury indicated use of the contralateral limb.

Specifically, subjects wore laboratory-provided athletic shoes to maintain standardized footwear and completed a 5-min warm up on a cycle ergometer (Lode, Groningen, Netherlands) at 50 W before all test sessions. Calibration was performed before each test session per manufacturer instructions. At the first preflight session, the dynamometer was fit to each subject, and position settings were recorded so that they could be replicated for future test sessions. An anatomic reference (knee = 90°, ankle = 0°, trunk = 0°) was measured with a hand-held goniometer during subject set-up for each joint tested. Knee testing was conducted in the seated position over a range of 95° (flexion) to 20° (extension). Ankle testing was performed prone over a subject’s maximal active range of motion rounded down to the nearest 5°. For example, if a subject could attain −18° of ankle flexion and 37° of ankle extension, range of motion was set at −15° (dorsiflexion) to 35° (plantarflexion). Trunk testing was conducted in the seated position from 0° (extension) to 90° (flexion). Testing was always performed in the order described below.

After cycle ergometer warm up, subjects performed five warm-up repetitions of knee extension/flexion (60°s^−1^, concentric/concentric) at 50% of their perceived maximum effort followed by two repetitions at 75–90% of maximum effort. After a 1- to 2-min rest, subjects performed three maximal knee extension/flexion repetitions. Subsequently, subjects performed three warmup repetitions of knee extension/flexion (180° s^−1^, concentric/concentric) at 50% of their perceived maximum effort followed by a 2-min rest. Then they completed 20 consecutive maximal repetitions (180° s^−1^, concentric/concentric) of knee extension/flexion. Ankle testing was performed in a similar manner. After an initial warm up (five repetitions at 50% of perceived maximum, two repetitions at 75–90% of maximum effort), subjects completed three maximal repetitions (30° s^−1^, concentric/concentric) of ankle extension (plantarflexion)/flexion (dorsiflexion). The final ankle tests were also ankle extension/flexion (30° s^−1^), but these tests were completed eccentrically with subjects maximally resisting the movement of the dynamometer. After one warm-up repetition at 50% of perceived maximal effort, subjects completed five maximal repetitions of discrete ankle plantarflexion followed by a set of five maximal repetitions of discrete ankle dorsiflexion. Last, subjects performed five warm-up repetitions of trunk flexion/extension (60° s^−1^, concentric/concentric) at 50% of their perceived maximum followed by two repetitions at 75–90% of maximum effort. After a 1- to 2-min rest, subjects performed three maximal trunk flexion/extension repetitions.

Subjects were instructed not to eat a large meal for at least 2 h before testing but could eat a light snack up to 1 h before testing. No nicotine or alcohol was allowed for 8 h before testing; caffeine was restricted to one cup of coffee or its caffeine equivalent which was permitted up to 1 h before testing. In addition, subjects could not perform a neutral buoyancy dive (training for extravehicular activity) for 72 h before testing, maximal exercise for 24 h before a scheduled evaluation, or any exercise 8 h before testing.

#### Muscle function

The functional fitness test (FFT) battery evaluates functional muscular strength, flexibility, and agility. The FFT was performed 60–90 days before flight; postflight testing was conducted 5–7 days after landing^32^. The following four measures were evaluated:

##### Leg press (1RM)

After a warm up at ~50% load for 10 repetitions on a leg press machine (Cybex International, Medway, MA), the load was increased 15–20% each set with decreasing repetitions until the subject could only complete 1 repetition at which point the load was increased 5–10% until failure. Subjects rested 3–5 min between sets. Leg press 1RM was recorded as the maximum weight successfully lifted.

##### Bench press (1RM)

After a warm up at ~30% load for 10 repetitions on a smith machine (Cybex International, Medway, MA), the load was increased 10–20% each set with decreasing repetitions until the subject could only complete 1 repetition at which point the load was increased 5–10% until failure. Subjects rested 3–5 min between sets. Bench press 1RM was recorded as the maximum weight successfully lifted.

##### Sit and reach

Lower back and hamstring flexibility was tested using an Acuflex I sit and reach box (Novel Products, Rockton, IL). Subjects were instructed to remove their shoes and place the feet against the footplate, then slowly reach forward, bending at the lumbar spine with knees in a fully extended position and hands one over the other. Subjects reached forward as far as possible while holding the most distant point momentarily. The score was recorded as the furthest reach of three trials.

##### Cone agility

Cone agility measured subjects’ ability to move quickly in four directions (forward, backward, left, and right) and to rapidly change directions^32^. Cones were placed at corners of a 4.57 m square and, starting at the lower left corner of the square, subjects were instructed to: (1) move forward to the upper left corner, (2) shuffle right to the upper right corner, (3) move backwards to the lower right corner, (4) shuffle left to the lower left corner (starting point), (5a) turn 45° to the right and move forward in a diagonal direction to the upper right corner, (5b) turn 135° to the left and move forward to the upper left corner, and (5c) turn 135° to the left and move forward to the lower right corner. The entire circuit was completed as quickly as possible; a hand-held stopwatch quantified the best of three trials.

#### Cardiorespiratory fitness

Aerobic fitness was evaluated during upright peak cycle ergometry tests (Lode Excalibur Sport; Lode B.V., Groningen, the Netherlands) performed twice before launch (L-180 d and L-50 d with the latter used for pre-flight baseline) and twice after return. Sprint subjects were tested on *R* + 1 and *R* + 30; most control subjects were tested on *R* + 3 and *R* + 30. The protocol consisted of a 3-min warm up at 50 W, followed by 1-min stepwise increments of 25 W to volitional fatigue. The HR and heart rhythm were monitored continuously (GE CASE, GE Healthcare, Chicago, IL). Ventilation and expired gas fractions (F_E_O_2_ and F_E_CO_2_) were measured continuously using the Portable Pulmonary Function System (PPFS), a metabolic gas analyzer commissioned by the European Space Agency and manufactured by the Danish Aerospace Company (DAC, Odense, DK)^35^. VO_2pk_ was defined as the highest 30-s average and was confirmed by the attainment of at least two of three criteria: (1) respiratory exchange ratio of > 1.09; (2) HR >90% of age-predicted maximum; (3) a plateau in VO_2_ (an increase of <150 mL min^−1^) from the previous stage. Ventilatory threshold was defined as the point at which VCO_2_ began to increase disproportionate to VO_2,_ and V_E_/VO_2_ increased with no comcomitant increase in V_E_/VCO_2_^46^.

#### Statistical analysis

Statistical analyses were conducted using Stata, IC software (v15.1) setting two-tailed alpha to reject the null hypothesis at 0.05. Our experimental design was a mixed-factorial with repeated observations collected preflight and postflight in which astronauts either participated in the Sprint intervention or the standard ISS exercise protocol. All outcomes were collected preflight and postflight with repeated postflight observations on some outcomes. We evaluated the effects of the Sprint exercise protocol (relative to standard of care) and spaceflight (preflight vs. each postflight) in separate mixed-effects models per dependent variable, with a priori simple interaction terms comparing each postflight to preflight, and simple interaction effects evaluating the relative change from preflight by group. Each of these models included a random y-intercept to accommodate the within-subjects experimental design, and degrees of freedom calculated per our repeated-measures experimental design. Each statistical test also underwent a rigorous examination of the distribution of model residuals before hypothesis testing and while nearly all of our analyses were satisfactory, it was necessary to use the inverse-cubic transformation for one outcome (cone test performance) to meet model assumptions, and to occasionally eliminate an overly influential observation (standardized residuals >3 and failure of the normality test). The data contained in this study constitute private medical information. As such, they are only available upon request in a deidentified fashion from NASA’s Life Sciences Data Archive (Life Sciences Data Archive).

### Reporting summary

Further information on research design is available in the Nature Research Reporting Summary linked to this article.

## Supplementary information

Supplementary Information

Reporting Summary

## Data Availability

Data from this study may be obtained through a data request in the NASA Life Science Data Archive (https://lsda.jsc.nasa.gov/Request/dataRequest). The study title “Integrated Resistance and Aerobic Training Study—Sprint” should be entered in the “Data Request Description”.
