# Supplementary material for: High intensity training during spaceflight: results from the NASA Sprint Study
Source: NPJ Microgravity. 2020 Aug 18;6:21. doi: 10.1038/s41526-020-00111-x (PMC7434884; doi:10.1038/s41526-020-00111-x)
Supplement: Supplementary file 1 — Supplementary Information [file 41526_2020_111_MOESM1_ESM.pdf]

Supplementary Table 1. Descriptive data for aerobic exercise training parameters on CEVIS and T2 during long-duration spaceflight with traditional exercise or Sprint exercise prescription

	CEVIS		T2	
	CON	SPRINT	CON	SPRINT
Volume (min•week^{-1})	78±33	55±15	87±31	81±27
30 s interval				
peak HR (b•min^{-1})	NA	165±9	156±14	167±15
peak HR (% maximum)	NA	86.4	90.8	93.1
2 min interval				
peak HR (b•min^{-1})	158±11	166±11	158±9	163±25
peak HR (% maximum)	92.3	92.7	91.7	90.9
4 min interval				
peak HR (b•min^{-1})	156±12	164±19	156±8	169±11
peak HR (% maximum)	90.8	90.6	89.0	93.8
Continuous				
peak HR (b•min^{-1})	151±12	151±11	145±16	154±21
peak HR (% maximum)	88.1	84.4	85.1	86.0

Data are mean±SD. CEVIS = cycle ergometer with vibration isolation system; T2 = second generation treadmill; CON = control group that performed the ISS standard of care exercise prescription; SPRINT = experimental group that performed a high intensity/lower volume exercise prescription; HR = heart rate.

Supplementary Table 2. Descriptive data for resistance exercise training parameters during long-duration spaceflight with traditional exercise or Sprint exercise prescription

	Squat		Heel Raise		Deadlift	
	CON	SPRINT	CON	SPRINT	CON	SPRINT
Load (kg)	89.9	95.3	115.8	133.3	85.3	91.3
Repetitions•week^{-1}	193.1	104.1	200.8	108.6	287.9	171.4
Volume load (kg•week^{-1})	17,014	9,613	22,946	13,871	22,917	15,074

Data are means. Volume load = sets × repetitions × load. "Squat" represents: back squat, single leg squat, and sumo squat; "heel raise" represents: heel raise and single leg heel raise; "deadlift" represents: deadlift, Romanian deadlift, and sumo deadlift. CON = control group that performed the ISS standard of care exercise prescription; SPRINT = experimental group that performed a high intensity/lower volume exercise prescription.

Supplementary Table 3. Sprint aerobic exercise prescriptions

Work time	Repetitions	Intensity (% VO_{2peak})	Rest
30 min	1	75	NA
4 min	4	90	3 min
2 min	6	70, 80, 90, 100, 90, 80	2 min
30 s	8	100	15 s

Supplementary Table 4. Sample 12-week Sprint resistance exercise prescription with exercises and load

	Day 1	Day 2	Day 3
**Exercises**	**Squat, Heel Raise, Romanian Deadlift**	**Sumo Squat, Heel Raise, Deadlift**	**Single-leg Squat, Heel Raise, Romanian Deadlift, Sumo Deadlift**
Week 1	Low	Low	Low
2	Low	Low	Low
3	Moderate	Low	High
4	High	Moderate	Low
5	Low	High	Moderate
6	Moderate	Low	High
7	High	Moderate	Low
8	Low	High	Moderate
9	Moderate	Low	High
10	High	Moderate	Low
11	Low	High	Moderate
12	Moderate	Low	High

Low load = 4 sets of 12 repetitions; Moderate load = 4 sets of 8 repetitions; High load = 4 sets of 6 repetitions. Sprint crewmembers performed resistance exercise 3 days/week. On a typical 6-month mission, crewmembers completed this 12-week cycle twice, although Weeks 1 and 2 (familiarization) were not repeated.

Supplementary Table 5. Sample 12-week resistance exercise prescription completed by control subjects with exercises and load

	Days 1,6,8	Days 2,4,9	Days 3,5,7
**Exercises**	**Squat, Heel Raise, Deadlift, Romanian Deadlift**	**Sumo Squat, Heel Raise, Deadlift**	**Single-leg Squat, Sumo Deadlift, Romanian Deadlift**
Weeks 1-3	Low	Low	Low
3-4	High	Low	Moderate
4-5	Moderate	High	Low
5-6	Low	Moderate	High
6-7	High	Low	Moderate
7-8	Moderate	High	Low
8-9	Low	Moderate	High
9-10	High	Low	Moderate
10-11	Moderate	High	Low
11-12	Low	Moderate	High
12	High	Low	Moderate

Low load = 4 sets of 12 repetitions; Moderate load = 4 sets of 8 repetitions; High load = 4 sets of 6 repetitions; heel raise was prescribed as 4 sets × 20 repetitions. Control crewmembers performed resistance exercise 6 days per week with a 9-day rotation. On a typical 6-month mission, crewmembers completed this 12-week mesocycle twice.
