# Supplementary material for: High intensity training during spaceflight: results from the NASA Sprint Study
Source: NPJ Microgravity. 2020 Aug 18;6:21. doi: 10.1038/s41526-020-00111-x (PMC7434884; doi:10.1038/s41526-020-00111-x)
Supplement: Supplementary file 2 — Reporting Summary [file 41526_2020_111_MOESM2_ESM.pdf]

nature research

Corresponding author(s): Kirk L. English

Last updated by author(s): Jul 8, 2020

Reporting Summary

Nature Research wishes to improve the reproducibility of the work that we publish. This form provides structure for consistency and transparency in reporting. For further information on Nature Research policies, see our Editorial Policies and the Editorial Policy Checklist.

Statistics

For all statistical analyses, confirm that the following items are present in the figure legend, table legend, main text, or Methods section.

n/a	Confirmed	
☐	☒	The exact sample size (*n*) for each experimental group/condition, given as a discrete number and unit of measurement
☐	☒	A statement on whether measurements were taken from distinct samples or whether the same sample was measured repeatedly
☐	☒	The statistical test(s) used AND whether they are one- or two-sided<br>*Only common tests should be described solely by name; describe more complex techniques in the Methods section.*
☒	☐	A description of all covariates tested
☐	☒	A description of any assumptions or corrections, such as tests of normality and adjustment for multiple comparisons
☐	☒	A full description of the statistical parameters including central tendency (e.g. means) or other basic estimates (e.g. regression coefficient) AND variation (e.g. standard deviation) or associated estimates of uncertainty (e.g. confidence intervals)
☐	☒	For null hypothesis testing, the test statistic (e.g. *F*, *t*, *r*) with confidence intervals, effect sizes, degrees of freedom and *P* value noted<br>*Give P values as exact values whenever suitable.*
☒	☐	For Bayesian analysis, information on the choice of priors and Markov chain Monte Carlo settings
☒	☐	For hierarchical and complex designs, identification of the appropriate level for tests and full reporting of outcomes
☒	☐	Estimates of effect sizes (e.g. Cohen's *d*, Pearson's *r*), indicating how they were calculated

Our web collection on statistics for biologists contains articles on many of the points above.

Software and code

Policy information about availability of computer code

Data collection	Dual Energy X-ray Absorptiometry (DXA) scans were obtained using a single densitometer (Hologic Discovery; Hologic Inc., Waltham, MA, USA). Isokinetic strength data were acquired for the knee, ankle, and trunk via a Biodex System 4 dynamometer (Biodex Medical Systems, Shirley, NY). A proprietary software package [Agile Data Analyzer and Monitor--ADAM] was used to compute metabolic gas analysis variables from metabolic testing.
Data analysis	Data were analyzed with Stata, IC software (v15.1).

For manuscripts utilizing custom algorithms or software that are central to the research but not yet described in published literature, software must be made available to editors and reviewers. We strongly encourage code deposition in a community repository (e.g. GitHub). See the Nature Research guidelines for submitting code & software for further information.

Data

Policy information about availability of data

All manuscripts must include a data availability statement. This statement should provide the following information, where applicable:

- Accession codes, unique identifiers, or web links for publicly available datasets
- A list of figures that have associated raw data
- A description of any restrictions on data availability

The data contained in this study constitute private medical information. As such, they are only available (upon request) in a de-identified fashion from NASA's Life Sciences Data Archive (LSDA).

Field-specific reporting

Please select the one below that is the best fit for your research. If you are not sure, read the appropriate sections before making your selection.

☒ Life sciences ☐ Behavioural & social sciences ☐ Ecological, evolutionary & environmental sciences

For a reference copy of the document with all sections, see nature.com/documents/nr-reporting-summary-flat.pdf

Life sciences study design

All studies must disclose on these points even when the disclosure is negative.

Sample size	Sample size for this study was determined by the NASA Human Research Program based on financial, programmatic, and timeline feasibility and practicality.
Data exclusions	Each statistical test also underwent a rigorous examination of the distribution of model residuals before hypothesis testing and while nearly all of our analyses were satisfactory, it was necessary to use the inverse-cubic transformation for one outcome (cone test performance) to meet model assumptions, and to occasionally eliminate an overly influential observation (standardized residuals >3 and failure of the normality test).
Replication	Due to the nature of this investigation, there were no attempts at replication.
Randomization	All National Aeronautics and Space Administration (NASA), Canadian Space Agency (CSA), European Space Agency (ESA), and Japan Aerospace Exploration Agency (JAXA) astronauts assigned to ISS flight were eligible to participate in this investigation. Subjects self-selected into one of two groups: 1) subjects that performed the experimental Sprint exercise program on the ISS (SPRINT) or 2) subjects that performed the standard individualized exercise program on the ISS (CON). This was necessary because of the voluntary nature of spaceflight research studies as well as crewmembers' reluctance to relinquish control of the fundamental nature of their exercise programs.
Blinding	Subjects were not blinded due to the nature of the exercise intervention.

Reporting for specific materials, systems and methods

We require information from authors about some types of materials, experimental systems and methods used in many studies. Here, indicate whether each material, system or method listed is relevant to your study. If you are not sure if a list item applies to your research, read the appropriate section before selecting a response.

Materials & experimental systems

n/a	Involved in the study	
☒	☐	Antibodies
☒	☐	Eukaryotic cell lines
☒	☐	Palaeontology and archaeology
☒	☐	Animals and other organisms
☐	☒	Human research participants
☒	☐	Clinical data
☒	☐	Dual use research of concern

Methods

n/a	Involved in the study	
☒	☐	ChIP-seq
☒	☐	Flow cytometry
☒	☐	MRI-based neuroimaging

Human research participants

Policy information about studies involving human research participants

Population characteristics	Experimental group subjects: n=9: 8M/1F, 48±7 y, 178±5 cm, 77.7±12.0 kg; control group subjects: n=17: 14M/3F, 46±6 y, 176±6 cm, 80.6±10.5 kg. All crewmembers completed standard pre-flight medical screening and received clearance from their flight surgeon before participating in the study.
Recruitment	After assignment to an ISS mission, astronauts were briefed on the purpose and nature of the study. If they chose to participate, they self-selected into either the control or experimental group. This was necessary because of the voluntary nature of spaceflight research studies as well as crewmembers' reluctance to relinquish control of the fundamental nature of their exercise programs.
Ethics oversight	The study was approved by the Institutional Review Board at NASA Johnson Space Center (JSC, Houston, TX), the Japan Aerospace Exploration Agency (JAXA) Institutional Review Board, the European Space Agency (ESA) Medical Board, and the Human Research Multilateral Review Board; all subjects provided written informed consent before participating in the study.

Note that full information on the approval of the study protocol must also be provided in the manuscript.
